# Retinoic acid signaling drives differentiation toward the absorptive lineage in colorectal cancer

**DOI:** 10.1016/j.isci.2021.103444

**Published:** 2021-11-15

**Authors:** Roelof A. Wester, Lisa van Voorthuijsen, Hannah K. Neikes, Jelmer J. Dijkstra, Lieke A. Lamers, Siebren Frölich, Maarten van der Sande, Colin Logie, Rik G.H. Lindeboom, Michiel Vermeulen

**Affiliations:** 1 Department of Molecular Biology, Faculty of Science, Radboud Institute for Molecular Life Sciences (RIMLS), Oncode Institute, Radboud University Nijmegen, 6525GA Nijmegen, the Netherlands; 2 Department of Molecular Developmental Biology, Faculty of Science, Radboud Institute for Molecular Life Sciences (RIMLS), Radboud University Nijmegen, 6525GA Nijmegen, the Netherlands; 3 Wellcome Sanger Institute, Wellcome Genome Campus, Hinxton, Cambridge CB10 1SA, UK

**Keywords:** Biological sciences, Cancer, Cell biology, Functional aspects of cell biology, Oncology

## Abstract

Retinoic acid (RA) signaling is an important and conserved pathway that regulates cellular proliferation and differentiation. Furthermore, perturbed RA signaling is implicated in cancer initiation and progression. However, the mechanisms by which RA signaling contributes to homeostasis, malignant transformation, and disease progression in the intestine remain incompletely understood. Here, we report, in agreement with previous findings, that activation of the Retinoic Acid Receptor and the Retinoid X Receptor results in enhanced transcription of enterocyte-specific genes in mouse small intestinal organoids. Conversely, inhibition of this pathway results in reduced expression of genes associated with the absorptive lineage. Strikingly, this latter effect is conserved in a human organoid model for colorectal cancer (CRC) progression. We further show that RXR motif accessibility depends on progression state of CRC organoids. Finally, we show that reduced RXR target gene expression correlates with worse CRC prognosis, implying RA signaling as a putative therapeutic target in CRC.

## Highlights

• RA signaling contributes to enterocyte differentiation in murine intestinal organoids
• Inhibition of RXR decreases enterocyte gene expression in colon cancer organoids
• Accessibility of RXR motifs correlates with RXRi susceptibility
• High expression of RA signaling targets correlates with higher CRC patient survival

## Graphical abstract

**Figure:**
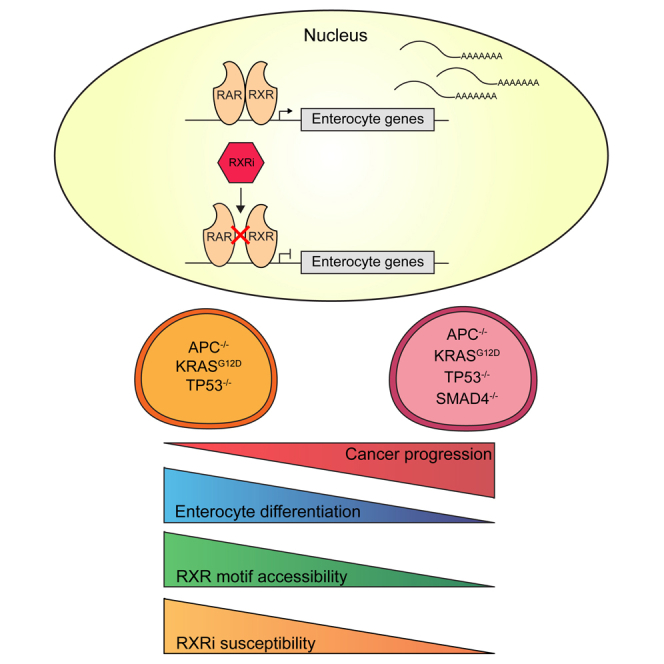

## Introduction

Vitamin A metabolism is essential in the human body. During digestion of nutrients, the intestinal epithelium absorbs vitamin A from the intestinal lumen (Theodosiou et al., 2010). Vitamin A, also known as retinol, is further processed via several enzymes to the effector molecule retinoic acid (RA), which exists in several stereoisomeric forms, such as all-trans retinoic acid (ATRA) and 9-cis retinoic acid (9-cis RA) (Chlapek et al., 2018). These compounds can bind retinoic acid receptor (RAR) and retinoid X receptor (RXR), respectively, which form heterodimers. These heterodimers interact with specific DNA sequences called nuclear hormone response elements (NREs). In their unliganded state, co-repressor proteins such as NCOR are recruited to nuclear hormone receptors bound to NREs, but these are exchanged for co-activator proteins such as NCOA2 in the presence of ATRA (Evans and Mangelsdorf, 2014). RA signaling is well known for its role in various biological processes such as differentiation, reproduction, embryogenesis, and eye development (Cunningham and Duester, 2015; Ghyselinck and Duester, 2019).

Loss of the nuclear hormone receptor RARα leads to aberrant lineage specification in the intestinal epithelium and is associated with increased Paneth and goblet cell numbers concomitant with a decreased number of enteroendocrine cells (Jijon et al., 2018). Furthermore, vitamin A is involved in intestinal immune homeostasis *in vivo,* which results in altered immunological responses upon vitamin A deficiency (Cassani et al., 2012). In previous work we showed that retinol metabolism is one of the most prominent upregulated metabolic pathways during enterocyte differentiation in mouse small intestinal organoids (Lindeboom et al., 2018), suggesting a role for RA signaling in intestinal stem cell differentiation toward the absorptive intestinal lineage. Interestingly, aberrant regulation of retinol metabolism is observed in colorectal cancer (CRC), and treating *Apc*^−/-^ mice with ATRA has been shown to decrease tumor initiation and metastasis (Ordóñez-Morán et al., 2015; Tang and Gudas, 2011). However, the molecular mechanisms underlying the effects of RA signaling on lineage specification and tumorigenesis in the intestine require further investigation.

Here, we studied the effects of perturbing RA signaling in mouse small intestinal organoids and in a human colon tumor progression organoid model (Drost et al., 2015). In mouse small intestinal organoids we observed a cystic phenotype, thinner crypt structures, and decreased enterocyte-specific gene expression upon RXR inhibition, whereas activation of RXR and RAR resulted in an opposite gene expression signature. Strikingly, the RA signaling-associated enterocyte differentiation phenotype is conserved in colon cancer organoids with three driver mutations (AKP), whereas more progressed human colon cancer organoids carrying four driver mutations (AKPS) are less sensitive to RA signaling perturbations. This is possibly due to the fact that RA signaling responsive genes are less accessible to RXR binding in AKPS compared with AKP organoids. Finally, we define RXR target genes, such as enterocyte markers *FABP1* and *SLC7A7*, and find that increased expression of these genes is associated with a better prognosis, suggesting that RA signaling could be a therapeutic target in CRC.

## Results

**Figure 1:**
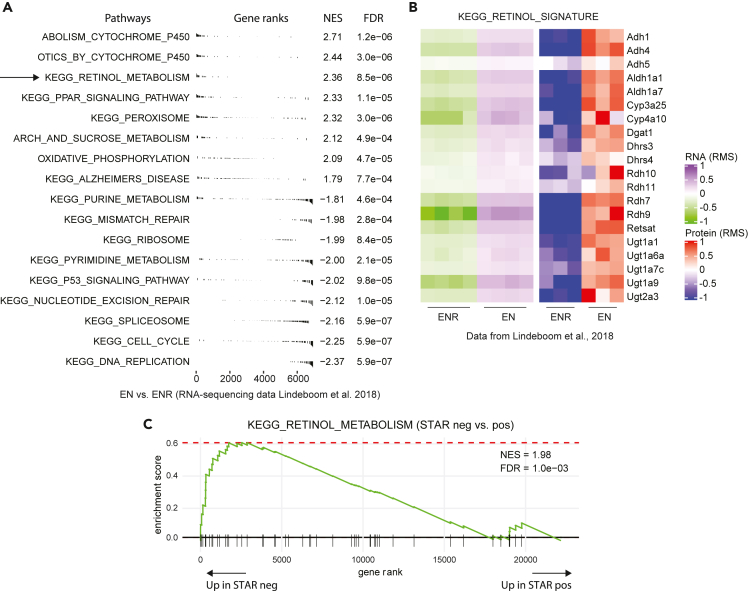
Retinol metabolism is enriched in differentiated cells of the intestine (A) Gene set enrichment analysis (GSEA) of gene expression dynamics in enterocyte-enriched mouse small intestinal organoid cultures (EN) versus wt organoids (ENR). Significantly enriched KEGG gene sets (false discovery rate [FDR] <0.001) associated with EN gene expression have a positive normalized enrichment score (NES), whereas gene sets associated with ENR gene expression have a negative NES. Genes associated with retinol metabolism are enriched in EN organoids. (B and C) (B) Expression of retinol metabolism-associated genes in ENR and EN organoids. The heatmaps are row-matched, and all the genes that are shown are dynamically expressed at the protein level. The left heatmap shows mRNA dynamics, whereas the right heatmap shows protein dynamics of the indicated genes. Relative expression is visualized as log2 fold change over the row mean (RMS). Genes associated with the retinol metabolism are upregulated in enterocyte-enriched organoids on RNA and protein levels. (C) GSEA of gene expression dynamics in STAR-negative versus STAR-positive cells of human colon and colorectal cancer organoids (Oost et al., 2018). STAR-negative cells represent differentiated cells and are significantly enriched for retinol metabolism-associated gene expression (NES = 1.98, FDR = 0.001).

Previously, we applied a multi-omics approach to study enterocyte differentiation in mouse small intestinal organoid cultures (Lindeboom et al., 2018). In this study we compared organoids grown in EN (EGF + Noggin) culture medium, which induces enterocyte differentiation, with organoids grown in standard ENR culture medium (EGF + Noggin + R-Spondin). Reanalysis of this multi-omics data of these organoid cultures revealed that both RNA and protein levels of genes involved in retinol metabolism are enriched upon enterocyte differentiation (Figures 1A and 1B). To further validate this finding, we interrogated transcriptomic data from human colon stem cells, isolated using a stem cell activity reporter (STAR) that we previously published (Oost et al., 2018). In this system, organoids were sorted for expression of an ASCL2-responsive fluorescent reporter. Retinol metabolism genes were enriched in STAR-negative cells (i.e., differentiated), again indicating that differentiation in the intestine is accompanied by increased retinol metabolism (Figure 1C). Taken together, these data are in accord with the notion that retinol metabolism plays a role in intestinal differentiation (Jijon et al., 2018; Lindeboom et al., 2018; Lukonin et al., 2020).

### Modulation of RA signaling drives cell fate switches in intestinal cells

**Figure 2:**
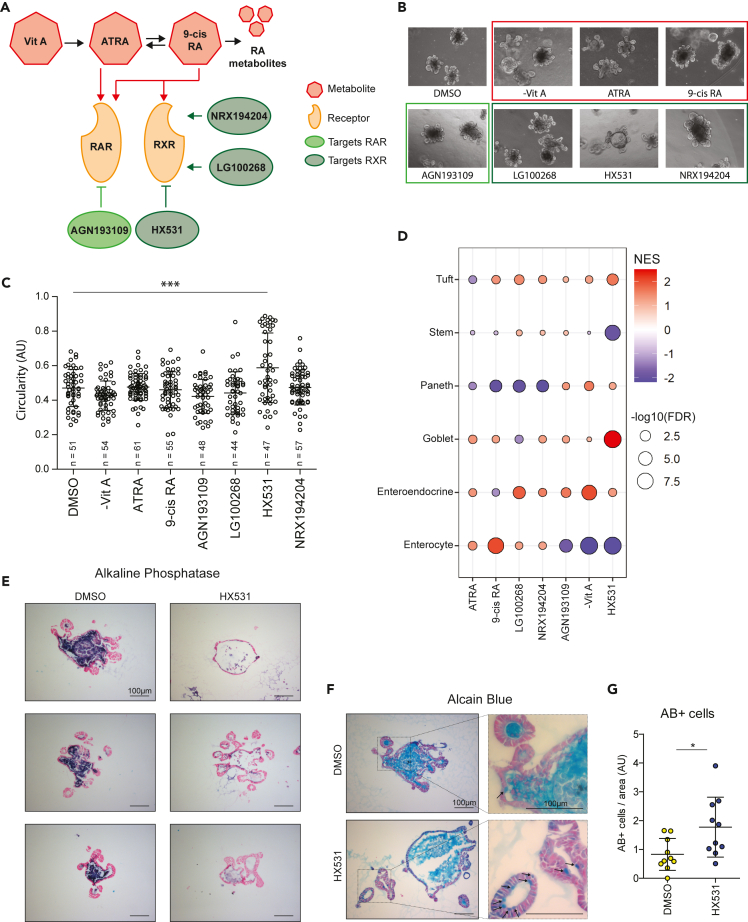
Modulation of RA signaling alters intestinal differentiation (A) Schematic overview of drugs used in this study. Biological ligands for RAR (ATRA) and RXR (9-cis RA) were used, as well as a synthetic RAR antagonist (AGN193109), a synthetic RXR antagonist (HX531), and two synthetic RXR agonists (LG100268, NRX1942014). (B) Bright-field microscopic photographs of mouse small intestinal organoids treated with drugs indicated in (A). HX531 treatment results in cystic organoids with thin, elongated crypts. Colored rectangles refer to colors used in (A). (C) Quantification of organoid circularity. HX531-treated organoids are more circular, meaning that their area to perimeter ratio is larger. Number of organoids measured is indicated in the plot; mean ± SD is shown. ∗∗∗p < 0.001 (ANOVA followed by Dunett's post-hoc test). Only HX531 significance is shown; comparisons of other treatments with control all result in p > 0.05. (D) Bubble plot showing GSEA parameters normalized enrichment score (NES, color) and FDR (size) for cell type-specific gene sets from Haber et al. (2017) in organoids treated with compounds indicated in (A). All enrichment scores were calculated taking DMSO as reference. Inhibition of either RAR or RXR leads to dramatic reduction of enterocyte-specific genes (n = 1 per condition). (E) Alkaline phosphatase/nuclear fast red staining for DMSO and HX531-treated organoids. Alkaline phosphatase expression is abundantly clear at the apical membrane and in the lumen of control organoids, but greatly reduced in HX531-treated organoids. Three images shown for each condition; scale bar, 100μm. (F) Alcian blue/nuclear fast red staining for DMSO and HX531-treated organoids. Goblet cells containing mucous are blue and have been marked with arrows. The amount of goblet cells in HX531-treated organoids is increased. Scale bar, 100μm. (G) Quantification of (F), Alcian blue-positive (AB+) cells over area (arbitrary units) is shown for DMSO and HX531-treated organoids. n = 10 images for each condition, mean ± SD is shown. ∗p < 0.05 (Mann-Whitney U test).

Inside cells, retinol can be converted to ATRA, which is the biological ligand for RAR (Chlapek et al., 2018). RAR can form a heterodimer with RXR and interact with nuclear hormone response elements in the genome to induce gene expression in an ATRA-dependent manner. To further investigate the role of retinol metabolism in intestinal organoids, we treated intestinal organoids with several compounds that affect RA signaling and RAR-RXR-mediated gene expression: the biological ligands for RAR (ATRA) and RXR (9-cis RA), as well as several synthetic retinoids, namely, AGN193109, an RAR antagonist (Agarwal et al., 1996); NRX194204 and LG100268, two RXR agonists (Lala et al., 1996; Vuligonda et al., 2001); and HX531, an RXR antagonist (Ebisawa et al., 1999; Morishita et al., 2009) (Figure 2A). RAR or RXR activation using either the natural agonists ATRA and 9-cis RA, or synthetic RXR agonists NRX194204 and LG100268, had little effect on organoid morphology. Inhibition of RXR homo- and heterodimers using HX531, however, yielded organoids that were cystic and were characterized by thin crypt structures (Figure 2B). We quantified the circularity of organoids and found that treatment with HX531 results in rounder structures (Figure 2C and S1C) (Thomas, 2019). We performed transcriptomic analysis on these organoids, and find that, although correlation coefficients are generally high, HX531-treated organoid cultures cluster away from other samples, indicating more striking differences (Figure S1A). Next, we selected cell-type-specific genes in the small intestine based on a recently published single-cell transcriptome dataset (Haber et al., 2017). We then investigated the effects of the treatments on this subset of genes, and observed that RAR and RXR agonists clustered together, and both induce increased expression of enterocyte-specific genes (Figure S1B). This finding independently confirms recently published data by Lukonin et al. (2020), who show that activation of RA signaling leads to premature enterocyte differentiation in mouse small intestinal organoids. Conversely, addition of RAR and RXR antagonists to the organoid culture resulted in reduced expression of enterocyte lineage genes and concomitant upregulation of genes that are specific to cell types of the secretory lineage (Figures 2D and S1B). Presence of enterocytes correlates with high alkaline phosphatase (AP) activity (Goldberg et al., 2008). Therefore, we performed an alkaline phosphatase staining on cryosections of organoids in the different treatments and observed a marked loss of AP activity upon HX531 treatment (Figures 2E and S1D). Interestingly, RXR inhibition resulted in increased expression of goblet cell-specific genes, whereas RAR inhibition specifically induced increased expression of genes that are specific for Paneth cells and the enteroendocrine lineage (Figure 2D). By staining acidic mucosubstances using Alcian blue (Scott, 1996), we show that inhibition of RXR using HX531 indeed results in an increased number of goblet cells (Figures 2F and 2G). Upregulation of secretory cells upon perturbation of enterocyte specification has been documented before (Chen et al., 2019; Lindeboom et al., 2018; Ueo et al., 2012). In summary, these results reveal that activation of RAR- and RXR-mediated gene expression drives enterocyte differentiation in the small intestine.

### Inhibition of RXR halts differentiation in colorectal cancer

**Figure 3:**
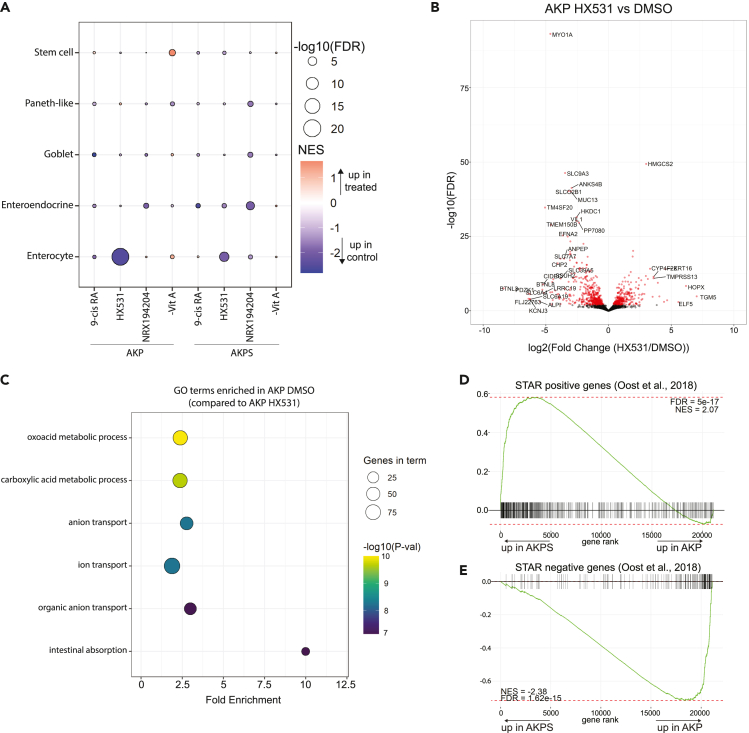
Inhibition of RXR disrupts differentiation in CRC organoids (A) Bubble plot showing GSEA parameters NES (color) and FDR (size) for cell type-specific gene sets from Wang et al. in AKP and AKPS organoids treated with indicated compounds. All enrichment scores were calculated using corresponding organoid line treated with DMSO as reference. Inhibition of RXR dimerization by HX531 treatment leads to severe reduction of enterocyte-specific genes in AKP and a modest reduction of these genes in AKPS organoids (n = 3 per condition). (B) Volcano plot showing genes that are differentially expressed in AKP control and HX531-treated organoids. Red dots indicate significantly differential genes (FDR <0.05, n = 740), most prominent differentially expressed (DE) genes are labeled. Among genes severely downregulated upon HX531 addition are several canonical enterocyte marker genes such as *ALPI* and *ANPEP*. (C) Bubble plot showing GO terms enriched in DMSO-treated organoids compared with HX531-treated organoids. Fold enrichment is plotted on the x axis; p value is indicated by color, and size of gene set by size. Intestinal absorption and other terms associated with mature enterocytes are significantly enriched in DMSO-treated organoids compared with HX531-treated organoids. (D) GSEA plot showing STAR-positive genes in AKP and AKPS. STAR-positive genes are significantly more enriched in AKPS, indicating that these organoids have more stem cells. (E) Same as (D), but for STAR-negative genes. These genes show enrichment in AKP, indicating that these organoids are more differentiated.

Induction of differentiation can be an effective therapeutic strategy in various malignancies. In line with the results we have presented thus far, RAR-RXR activation can result in decreased cancer cell proliferation and disease severity (Applegate and Lane, 2015; Modarai et al., 2018). These anti-cancer effects of RA signaling, in combination with its differentiation-associated phenotype in mouse small intestinal organoids, led us to hypothesize that RA signaling might also induce differentiation in progressed human CRC. To investigate this, we applied an organoid-based model for CRC progression. We specifically made use of isogenic human colon organoid lines carrying three or four oncogenic driver mutations, respectively (AKP: *APC*^−/-^, *KRAS*^G12D^, *TP53*^−/−^ and AKPS: *APC*^−/-^, *KRAS*^G12D^, *TP53*^−/−^, *SMAD4*^−/−^) (Drost et al., 2015). AKP organoids, when injected into nude mice, give rise to non-invasive adenomas, whereas AKPS organoids cause metastatic disease (Fumagalli et al., 2017). As our experiments thus far showed most prominent effects when we perturbed RXR, rather than RAR, we decided to limit further experiments to this receptor. We treated these organoid cultures with medium depleted for vitamin A or medium supplemented with 9-cis RA (biological RXR/RAR agonist), NRX194204 (synthetic RXR agonist), and HX531 (synthetic RXR antagonist) and performed RNA sequencing. First, we observed that most variation between samples can be explained by genotype (Figure S2A). Furthermore, treatment with HX531 induces major changes in gene expression in both AKP and AKPS, whereas activation of RXR with either natural or synthetic ligands does not. This latter observation is in contrast to a previous study, in which RA treatment in *Apc*^−/-^ mice led to decreased tumor initiation and metastasis (Ordóñez-Morán et al., 2015). This discrepancy may be explained by a context-specific lack of response upon RA treatment, due to the more advanced cancer progression status of our model system. We then extracted cell-type-specific gene sets from a recently published single-cell RNA sequencing (scRNA-seq) survey of the human intestine and colon (Wang et al., 2020). Using these gene sets, we found that both AKP and, to a lesser extent, AKPS organoids become depleted for enterocyte-specific genes upon RXR inhibition (Figure 3A). This reduction of enterocyte-specific gene expression is not compensated for by an increase in gene expression of any other mature intestinal cell type, which is in contrast to what we observed in the mouse small intestine. Furthermore, whereas mouse organoids showed marked differences in expression of enterocyte genes upon activation of RXR and depletion of vitamin A, CRC organoids do not. In AKP organoids, HX531 treatment results in reduced expression of several mature enterocyte marker genes, such as *ALPI* and *ANPEP* (Figure 3B). Gene Ontology (GO) analysis revealed that HX531 treatment, which blocks dimerization of RXR and its binding partners, led to depletion of terms associated with intestinal absorption and mature enterocyte function (Figure 3C). This treatment simultaneously led to an increase of GO terms associated with development of non-colonic epithelial tissues (Figure S2B). The latter phenomenon has also been observed when treating mouse small intestinal organoids with RXR antagonists (Lukonin et al., 2020). These findings indicate that dimerization of RXR is necessary for proper enterocyte specification.

In contrast to AKP, AKPS organoids do not contain a functional SMAD4 protein, which effectively inactivates TGFβ and BMP signaling pathways. The latter induces differentiation toward both the absorptive and the secretory lineage in the intestine (Auclair et al., 2007; Chen et al., 2019; Gehart and Clevers, 2019; Qi et al., 2017). By comparing AKP and AKPS expression profiles to those of an ASCL2-dependent STAR organoid line (Oost et al., 2018), we found that AKPS organoids are enriched for STAR-positive genes (i.e., stem cell genes) and that AKP organoids are enriched for STAR-negative genes (i.e., differentiation genes) (Figures 3D and 3E). This finding indicates that AKP is more differentiated, and perhaps therefore more susceptible to inhibition of differentiation by treatment with HX531. Taken together, these results show that inhibition of RA signaling reduces differentiation in CRC, and that this effect is more pronounced when cells undergoing treatment are more differentiated.

### RXR-mediated gene expression induces differentiation in colorectal cancer organoids

**Figure 4:**
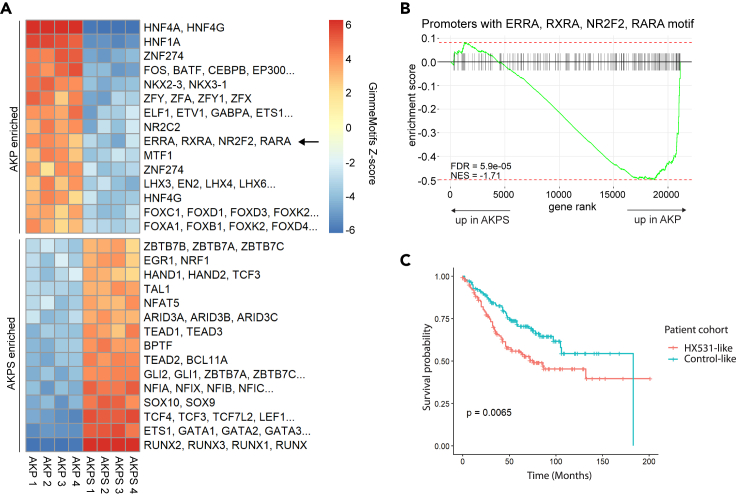
RXR-mediated differentiation disarms colorectal cancer (A) Heatmap displaying GimmeMotifs (maelstrom) *Z* score using differential ATAC-seq peaks (FDR<0.05). Higher *Z* score indicates that a motif is more accessible in a given sample. Differentiation-associated motifs such as HNF4A and HNF4G are more accessible in AKP. A motif that can be bound by RXRA (arrow) is also more accessible in AKP. (B) GSEA plot showing enrichment of genes that have an RXRA motif in their promoter in AKP and AKPS. Promoters were defined as 500 bp surrounding transcription start site. The sequence logo of the motif that was used is shown in Figure S3A. These genes are significantly enriched in AKP, indicating that accessibility of the motif corresponds to higher RNA expression. (C) Kaplan-Meier curve showing survival probability of patients with colorectal cancer based expression of selected genes. A g set was used composed of all genes that were significantly downregulated (p value < 0.01) in either AKP or AKPS organoids upon treatment with HX531. Blue line shows the 25% of patients with the highest expression of these genes, termed “Control-like.” Red line shows the 25% of patients with the lowest expression of these genes, termed “HX531-like.” Patients with HX531-like gene expression have significantly lower survival probability than patients with Control-like gene expression.

Thus far, we have shown that RA signaling induces differentiation in mouse small intestinal organoids, as well as in human CRC organoids, and that organoid cultures without active BMP/TGFβ signaling are less susceptible to RA signaling perturbations. To investigate other factors that contribute to the difference in differentiation status between the two cancer organoids, we performed assays for transposase-accessible chromatin by sequencing (ATAC-seq) (Buenrostro et al., 2013), which can be used to profile accessible regions in the genome. We mined the accessible regions in the AKP and AKPS genome and performed differential motif analysis using GimmeMotifs (Bruse and van Heeringen, 2018; van Heeringen and Veenstra, 2011). We observe that regions enriched in AKPS organoids contain RUNX and TCF4 motifs. Both factors have been implicated in metastasis (Figure 4A) (Li et al., 2019; White et al., 2012). Conversely, well-known intestinal differentiation-associated motifs such as HNF4A and HNF4G are enriched in AKP (Chen et al., 2019; Lindeboom et al., 2018). Importantly, we also observed enrichment of an RXRA/RARA motif in AKP (Figure 4A). Closer inspection of this motif revealed it to be very similar to a direct repeat (DR) half-site (AGGTCA) (Figure S3B) (Evans and Mangelsdorf, 2014). The number of nucleotides separating two tandem DRs (called DR1 through DR5) is believed to select for different NR heterodimers. However, we did not detect enrichment of a specific DR element, perhaps indicating that multiple RXR heterodimers function in concert. We then asked whether the differential accessibility of these motifs correlates with gene expression changes between AKP and AKPS. To that end, we took assigned accessible regions to their closest gene, filtered for differentially expressed genes (false discovery rate <0.05), and scanned for known motifs. This analysis revealed that regions associated with AKP-specific genes contain HNF4A, HNF4G, and RXRA motifs (Figures S3A, S3C, and S3D). It should be noted that we did not retrieve the same RXRA motif that was enriched in ATAC peaks; however, upon inspection we conclude that this motif constitutes two DR halfsites with one nucleotide spacing (Figures S3A and S3C). We then scanned promoters of all genes specifically for the DR half-site motif (Figure S3A). Gene set enrichment analysis showed significant enrichment of these genes in AKP, indicating that accessibility of this motif contributes to expression of genes associated with it (Figure 4B). Taken together, we conclude that RXR-mediated gene expression contributes to differentiation status in CRC organoids.

Finally, to investigate the potential clinical significance of these findings, we selected the genes that were downregulated upon inhibition of RXR and determined whether their expression impacts CRC patient survival (Marisa et al., 2013). This analysis revealed that patients displaying low expression of genes that are downregulated upon HX531 treatment (HX-like) in CRC organoids have worse survival probability than patients who show high expression of those genes (control-like) (Figure 4C). These findings suggest that RXR-dependent expression of differentiation-associated genes in the intestine contributes to a better prognosis and imply that RA signaling is a putative therapeutic target in CRC.

## Discussion

In this study, we find that retinol metabolism and RXR are linked to differentiation-associated gene expression in the murine small intestine and the human (malignant) colon. These observations are in line with recently published evidence for premature enterocyte differentiation in small intestinal organoids upon RAR-RXR activation (Lukonin et al., 2020), data that show decreased colon tumor initiation and metastasis upon RA treatment (Ordóñez-Morán et al., 2015), as well as with findings in other tissues concerning the differentiation-inducing capacity of RA signaling (Janesick et al., 2015; Mezquita and Mezquita, 2019; Theodosiou et al., 2010). Recent work by Lukonin and colleagues also implicates RA signaling in enterocyte differentiation (Lukonin et al., 2020). In that study, the authors observe downregulation of markers for all mature lineages of the small intestine upon inhibition of RXR. We used gene sets recently generated in an scRNA-seq survey of the small intestine (Haber et al., 2017) to determine that, upon RXR inhibition using HX531, enterocyte-specific genes are downregulated, whereas goblet cell-specific genes are upregulated (Figures 2C and S1B). Interestingly, disruption of other factors that drive absorptive lineage specification, such as HNF4G and HES1, results in a similar phenotype (Chen et al., 2019; Lindeboom et al., 2018; Ueo et al., 2012). Taken together with the fact that *Rxra* is one of nine enterocyte-specific transcription factors (Haber et al., 2017), this indicates that RXR is a bona fide driver of enterocyte differentiation.

As we previously observed increased retinol metabolism in enterocytes and given the well-documented role of RA signaling in cellular differentiation, we hypothesized that RAR-RXR-mediated gene expression regulation plays an important role in regulating cellular homeostasis and cell fate switches in the intestinal epithelium. In line with this hypothesis, perturbation of RAR-RXR signaling affects the expression of enterocyte-specific genes in mouse small intestinal and human CRC organoids. In mouse organoids, these effects were more pronounced when targeting RXR using 9-cis RA than ATRA, perhaps because 9-cis RA can activate both RAR and RXR (Evans and Mangelsdorf, 2014). Even though we find enterocyte-specific gene expression to be perturbed in both mouse and human organoids, we also observed several discrepancies. For example, we observed a different response upon activation of RA signaling using 9-cis RA in small intestinal organoids compared with human CRC organoids. Furthermore, whereas HX531 treatment leads to a marked increase of goblet cell-specific genes in the mouse small intestine, this effect is not observed in CRC organoids. These effects could be due to species or organ differences, or due to the presence or absence of cancer driver mutations, for example.

Despite our focus on RAR-RXR signaling, it should be emphasized that neither our motif analysis nor our inhibitor treatment in CRC organoids excludes other NRs from being involved in this process. In fact, there are several indications that other RXR binding partners fulfill critical roles in intestinal homeostasis and are linked to malignant transformation. First, depletion of vitamin A in CRC organoids and activation of RXR fail to capture the phenotypic severity of HX531 treatment, which blocks all forms of RXR dimerization. In addition, the vitamin D receptor has been shown to be a marker for stem cells in the murine intestine (Fernández-Barral et al., 2020; Haber et al., 2017). Furthermore, vitamin D deficiency is associated with an increased risk of CRC (Feldman et al., 2014). Recent evidence, however, has revealed that its activity is disease context dependent (Fernández-Barral et al., 2020). Similarly, the identification of differentially accessible thyroid hormone receptor α/β (THRA/THRB) motifs in AKP and AKPS organoids hints to a disease context-dependent activity of thyroid hormone in CRC. This idea is further supported by evidence concerning the role of thyroid hormone in CRC progression, with studies concluding that it limits tumor growth through differentiation (Cicatiello et al., 2017), whereas others report that it enhances tumor growth through repression of Wnt inhibitors (Uchuya-Castillo et al., 2018). Here, we report context-dependent effects of RXR inhibition. Although AKP organoids show a pronounced differentiation defect upon RXR inhibition, more progressed AKPS organoids are less susceptible to this treatment. Our findings imply that differences in the epigenetic landscape and genome accessibility could underlie these context-dependent effects. Taken together with findings on other NRs in cancer, this result indicates that efficacy of any potential therapeutic strategy targeting these proteins will be highly dependent on underlying oncogenic mutations and the associated chromatin architecture.

When examining potential explanations for this discrepancy in treatment efficacy, we found that overall AKP organoids express more differentiation-associated genes than AKPS. This is in line with the well-documented role of the BMP pathway, which is disrupted in AKPS organoids, in the intestine as an inducer of differentiation (Gehart and Clevers, 2019). For example, the BMP inhibitor Noggin is essential for maintenance of intestinal organoid cultures *in vitro* (Sato et al., 2009). Along with a more differentiated transcriptome, we found that the motif for HNF4G, a recently uncovered master regulator of enterocyte differentiation (Lindeboom et al., 2018), was the most enriched motif in AKP. Together with our findings pertaining to the differentiation-inducing effects of RA signaling, this alludes to the possibility that these factors might act in concert. Such concerted action has recently been described for HNF4G and SMAD4 (Chen et al., 2019). HNF4G only functions in homodimers (Jiang et al., 1995) and is therefore unable to function as an RXR dimerization partner, but it is possible that RAR-RXR and HNF4G strengthen each other's differentiation-inducing effects. It would be interesting to combine (conditional) knockouts and genomic localization studies, to disentangle these mechanisms.

In summary, in this study, we described a conserved differentiation-inducing effect of RA signaling toward the enterocyte lineage in mouse small intestinal and human colon organoids. This effect can be disrupted through inhibition of RXR dimerization and is at least partly dependent on a permissive chromatin structure. Integration of these findings with gene expression data in patients with CRC revealed that disruption of RXR-dependent induction of differentiation is associated with a poor prognosis. This demonstrates the clinical relevance of our findings and suggests that RA signaling is a therapeutic target in CRC.

### Limitations of the study

Organoids typically harbor several distinct cell types in a spatial organization that is phenotypically and functionally reminiscent of *in vivo* structures, making them attractive model systems compared with 2D cultures. Intestinal organoid cultures are composed of epithelial cells, but do not contain mesenchymal cells, meaning that several important niche cell types and factors are absent, which could negatively affect cellular maturation and/or differentiation. Furthermore, transcriptome and DNA accessibility assays were performed in bulk cultures, meaning that heterogeneous populations of cells were measured. Although these assays give valuable insights into the effect of perturbations on the organoid cultures, effects on prevalent cell types in the organoids could potentially mask effects in rare cell types. Performing these assays with single-cell resolution would address these issues.

## STAR★Methods

### Key resources table

**Table:** 

REAGENT or RESOURCE	SOURCE	IDENTIFIER
**Chemicals, peptides, and recombinant proteins**		

Cultrex RGF BME Type 2 PathClear	R&D Systems	Cat#3533-005-02
Advanced DMEM/F-12	Gibco	Cat# 12634028
Cultrex Organoid Harvesting Solution	R&D Systems	Cat#3700-100-01
Recovery Cell Culture Freezing Medium	Gibco	Cat# 12648010
N-Acetylcysteine	Sigma-Aldrich	Cat#A9165
HEPES	Sigma-Aldrich	Cat#H0887
GlutaMax	Gibco	Cat# 35050038
Penicillin-Streptomycin	Gibco	Cat# 15140122
Mouse EGF Recombinant Protein	Gibco	Cat#PMG8041
Nicotinamide	Sigma-Aldrich	Cat#72340
A83-01	Cayman Chemical	Cat# 9001799
SB202190	Cayman Chemical	Cat#10010399
B27 supplement	Gibco	Cat#17504044
B27 supplement (minus Vitamin A)	Gibco	Cat#12587010
Tn5 enzyme	In-house made	NA
Alcian Blue	Sigma-Aldrich	Cat#A3157
Nuclear Fast Red	Serva	Cat#26933
Alkaline Phosphatase	Vector Laboratories	Cat#AK-5000
Aluminium Sulfate Hydrate	Sigma-Aldrich	Cat#368458
Gluteraldehyde	Sigma-Aldrich	Cat# 340855
Tissue-Tek O.C.T. Compound	Sakaru	Cat# 4583
All-trans Retinoic Acid	Cayman Chemical	Cat# 11017
Dimethyl Sulfoxide (DMSO)	Sigma-Aldrich	Cat#317275
9-cis-Retinoic Acid	Cayman Chemical	Cat#14587
AGN 193109	Sigma-Aldrich	Cat#SML2034
NRX 194204	Axon	Cat#2408
HX531	Sigma-Aldrich	Cat#SML2170
LG100268	Sigma-Aldrich	Cat#SML0279

**Critical commercial assays**		

KAPA HiFi HotStart ReadyMix	KAPA Biosystems	Cat#KK2602
NextFlex DNA barcodes	PerkinElmer	Cat#NOVA-514106
Agencourt AMPure XP	KAPA Biosystems	Cat#07983280001
RNeasy RNA extraction kit	Qiagen	Cat# NC9307831
High Sensitivity DNA Bioanalyzer	Agilent	Cat# 5067-4626
DeNovix dsDNA High Sensitivity Assay	DeNovix	Cat#KIT-DSDNA-ULTRA-E

**Deposited data**		

RNA-seq	Lindeboom et al., 2018	GEO: GSE114113
RNA-seq, ATAC-seq	This study	GEO: GSE163142

**Experimental models: Cell lines**		

Lgr5-GFP-DTR mouse small intestinal organoids	Genentech	NA
AKP^APC-/-,TP53-/-,KRAS(G12D)^ human colon organoids	Stichting Hubrecht Organoid Technology	NA
AKP^APC-/-,TP53-/-,KRAS(G12D),SMAD4-/-^ human colon organoids	Stichting Hubrecht Organoid Technology	NA
Human 293T mNoggin cell line to make Noggin CM	J. den Hertog, Hubrecht Institute	NA
Human 293T RSpondin1 cell line to make R-Spondin CM	C. Kuo, Stanford University	NA

**Software and algorithms**		

Adobe Illustrator	Adobe	https://www.adobe.com/products/illustrator.html
FIJI	ImageJ	https://imagej.net/software/fiji/
Seq2Science	Van der Sande et al., 2020	NA
Prism (Version 5.03)	Graphpad	https://www.graphpad.com/scientific-software/prism/
RStudio	RStudio	https://www.rstudio.com/
DESeq2	Love et al., 2014	NA
FGSEA	Korotkevich et al., 2019	NA
GimmeMotifs	Bruse and van Heeringen, 2018	https://github.com/vanheeringen-lab/gimmemotifs

### Resource availability

#### Lead contact

Further information and requests for resources and reagents should be directed to and will be fulfilled by the lead contact, Michiel Vermeulen (Michiel.vermeulen@science.ru.nl).

#### Materials availability

This study did not generate new unique reagents.

### Experimental model and subject details

#### Mouse small intestinal organoid cultures

Female Mouse *Lgr5*^GFPDTR/+^ small intestinal organoids (Tian et al., 2011) were embedded in a mix of 90% ice-cold RGF BME Type 2 PathClear (Cultrex, R&D Systems) and 10% Dulbecco’s modified Eagles Medium/F12 (Gibco), and left at 37°C to allow BME to polymerize. Organoids were cultured in ENR medium (advanced Dulbeccos modified Eagles Medium/F12 (Gibco) supplemented with 1× Penicillin-Streptomycin (Gibco), 10 mM HEPES (Gibco), and 1× GlutaMAX™ Supplement (Gibco)), 1× B27 (Gibco) or 1× B27 without Vitamin A (Gibco), 1,25 mM N-acetylcysteine (Sigma Aldrich), 50 ng/mL mEGF (Gibco), 10% final volume noggin conditioned medium, 5% final volume R-Spondin1 conditioned medium) at 37°. For maintenance, organoids were split by mechanical shearing using a Pasteur pipette every 5 days and the culture medium was refreshed every 2–3 days (Sato et al., 2009).

#### Colon cancer organoid cultures

Female AKP^APC−/−,TP53−/−,KRAS(G12D)^ and AKPS^APC−/−,TP53−/−,KRAS(G12D)/SMAD4−/−^ organoids (Drost et al., 2015) were embedded in a mix of 90% ice-cold RGF BME Type 2 PathClear (Cultrex, R&D Systems) and 10% Dulbecco's modified Eagles Medium/F12 (Gibco), and left at 37°C for 30 minutes to allow BME to polymerize. Organoids were cultured in media containing advanced Dulbecco's modified Eagles Medium/F12 (Gibco) (supplemented with 1× Penicillin-Streptomycin (Gibco), 10 mM HEPES (Gibco), and 1× GlutaMAX™ Supplement (Gibco)), 1× B27 (Gibco) or 1× B27 without Vitamin A (Gibco), 1,25 mM N-acetylcysteine (Sigma Aldrich), 50 ng/mL mEGF (Gibco), 10% final volume noggin conditioned medium, 10 mM nicotinamide (Sigma Aldrich), 500 nM A83-01 (Cayman Chemical), and 10 μM SB202190 (Cayman Chemical). Note that Noggin was added to both AKP and AKPS organoid cultures. For maintenance, organoids were split by mechanical shearing using a Pasteur pipette every 5 days and the culture medium was refreshed every 2–3 days (Drost et al., 2015).

### Method details

#### Drug treatment

24 hours after splitting, organoids were treated with either ENR – Vitamin A containing 0.1% final volume DMSO, ENR + Vitamin A containing 0.1% final volume DMSO 5μM all-trans-Retinoic acid (ATRA, Cayman Chemical), 10μM 9- cis-Retinoic acid (9-cisRA, Cayman Chemical), 1μM AGN193109 (Sigma Aldrich), 1μM NRX194204 (Axon Medchem), 1μM HX531 (Sigma Aldrich) or 10 μM LG100268 (Sigma Aldrich). Drugs were stored under reduced light conditions and exposure to light was minimized. Media containing compounds was refreshed after 24h. Total treatment time was 48h for all drugs. All drugs were dissolved in DMSO and added to the culture medium at working concentration in 0.1% final volume DMSO. After treatment, organoids were harvested using Organoid Harvesting Solution (Cultrex, R&D Systems) and pelleted. Pellets were snap frozen and stored at −80 C for RNA-sequencing, or cryopreserved to maintain native chromatin context for ATAC-sequencing.

#### Histology and staining of organoids

Organoids in BME were fixed in 4% paraformaldehyde (PFA) and 0.5% glutaraldehyde in PBS for 15 min at room temperature. BME domes were incubated in 20% sucrose for 72 h at 4°C until they sank, after which sucrose was removed and domes were embedded in Tissue-Tek O.C.T. Coumpound (Sakaru) compound and snap-frozen. Cryotome sections of 6 μm thickness were subjected to histological assays. Acidic mucosubstances from goblet cells were detected with Alcian blue solution (1 g of Alcian blue 8GX (Sigma Aldrich), pH 2.5, 3 ml/l of acetic acid, and 97 ml of distilled water). After 30-min incubation, Nuclear Fast Red (0.1 g of Nuclear Fast Red (Serva), 5 g of aluminum sulfate hydrate (Sigma Aldrich), and 100 ml of distilled water) was used to counterstain. Alkaline Phosphatase was detected using Alkaline Phosphatase Substrate (Vector Laboratories) according to protocol and counterstained with Nuclear Fast Red. Organoid section images were taken by bright field microscopy using an AxioCam MRc5 microscope (Zeiss).

#### RNA-sequencing

RNA from snap frozen pellets of drug treated and control organoids was extracted using the RNeasy RNA extraction kit (Qiagen) with DnaseI treatment. A total amount of 1 μg RNA per mouse small intestinal sample or 100ng RNA per CRC organoid sample was used for the preparation of RNA sequencing libraries using the KAPA RNA HyperPrep Kit with RiboErase (HMR) (KAPA Biosystems). In short, oligo hybridization and rRNA depletion, rRNA depletion cleanup, Dnase digestion, Dnase digestion cleanup, and RNA elution were performed according to protocol. Fragmentation and priming were performed at 94 C for 6:30 min. First strand synthesis, second strand synthesis, and A- tailing was performed according to protocol. For the adapter ligation, a 7 μM stock was used (NextFlex DNA barcodes, Bioo Scientific). First, and second post-ligation cleanup was performed according protocol. For the library amplification, 6 cycli were used. The library amplification cleanup was performed using a 0.8x bead-based cleanup. Library size was determined using the High Sensitivity DNA bioanalyzer (Agilent Technologies), library concentration was measured using the DeNovix dsDNA High Sensitivity Assay (DeNovix). Sequencing was performed using an Illumina NextSeq 500, 50-bp paired-end reads were generated.

#### ATAC-sequencing

ATAC-seq was performed on roughly 50,000 cells of AKP and AKPS organoids in four technical replicates. Briefly, organoids were harvested using Organoids Harvesting Solution (Cultrex, R&D Systems), and cryopreserved in Recovery Cell Culture Freezing Medium (GIBCO) at -80°C. At the start of the experiment, cells were thawed quickly at 37°C and washed twice with PBS. Nuclei isolated in hypotonic buffer were tagmented for 5 minutes with in-house generated Tn5 while shaking at 37°C and DNA was purified using 2x SPRI beads (Ampure) purification. DNA was subsequently PCR amplified by using KAPA HiFi Hotstart Ready Mix (KAPA Biosystems) and Nextera Index Kit (Illumina) primers followed by reverse phase 0.65 x SPRI beads purification and a QIAquick Spin Column (QIAGEN) purification. Amplified DNA libraries were sequenced with an Illumina NextSeq 500 at a read length of 38 bp.

#### Image analysis

Images of single organoids were fed to a FIJI macro that measures several spatial parameters of a given object (Thomas, 2019). Threshold was determined individually to make sure the complete organoid was considered, watershed mode was disabled. Circularity, which is given by the formula 4π(area/perimeter^2^) was plotted for each treatment. We performed an ANOVA test followed by Dunett’s post-hoc test to assess significance. Alcain Blue positive cells were counted manually. We performed a Mann-Whitney U-test to assess significance. Details for statistical analysis are given in figure legends.

#### RNA-sequencing data analysis

RNA-seq data was processed using seq2science using default options (Van der Sande et al., 2020). In short, reads were first trimmed using Fastp. Sequencing reads were aligned to the mm10 (mouse) or hg38 (human) transcript assembly from UCSC using STAR, after which a count table was generated using HTSeq. For experiments without replicates (mouse experiments), counts per million reads were calculated for each sample and any transcript not detected in all samples was filtered, log2 values were calculated and genes were ranked. For experiments with replicates (human), differential gene expression analysis was performed with the DESeq2 package (Love et al., 2014). The ‘rlogTransformation’ function in DESeq2 was used to normalize, log2 transform and noise-stabilize the expression data for visualization purposes. Heatmaps for expression data were created with the ComplexHeatmap package (Gu et al., 2016). All pairwise comparisons have been made using control samples from the same organoid batch.

#### Gene set enrichment analysis

Gene set enrichment analysis was performed using the fgsea package (Korotkevich et al., 2019). Genes were ranked according to fold change calculated using the lfcShrink (apeglm) (Zhu et al., 2018) option in DESeq2. Mouse cell type specific gene sets were extracted from (Haber et al., 2017), human cell type specific gene sets from (Wang et al., 2020). STAR negative and positive gene sets were extracted from (Oost et al., 2018).

#### Survival analysis

Overall survival and expression data was used for survival analysis (Marisa et al., 2013). We defined a gene set containing genes significantly downregulated by HX531 compared to DMSO in AKP and AKPS (p<0.01). The mean z-score normalized expression of this gene signature in each colorectal cancer patient was used to compare the 25% highest expressing patients versus the 25% lowest.

#### ATAC-sequencing analysis

Preprocessing of reads was done automatically with workflow tool seq2science v0.3.0 (Van der Sande et al., 2020). Reads were aligned to hg38 with bwa-mem v0.7.17 (Li, 2013) with option '-M' to discard reads mapping to the mitochondrial chromosome. Mapped reads were removed if they did not have a minimum mapping quality of 30, were a (secondary) multimapper or aligned inside the ENCODE blacklist (Amemiya et al., 2019) and finally were tn5 bias shifted by seq2science. Afterwards, duplicate reads were removed with picard MarkDuplicates v2.21.2 (Picard, 2019). Next, paired-end info was removed from reads with seq2science to utilize both mates during peak calling with macs2 v2.2.7 (Zhang et al., 2008) with options '--shift -100 --extsize 200 --nomodel --keep-dup 1 --buffer-size 10000' in BAM mode. The effective genome size was estimated by taking the number of unique kmers in the assembly of the same length as the average read length for each sample. Narrowpeak files of replicates belonging to the same condition were merged with fisher's method in macs2 v2.2.7. Raw counts for all samples under each peak, as determined by GimmeMotifs (Bruse and van Heeringen, 2018) coverage_table function, were used to define differentially accessible regions with DESeq2 (Love et al., 2014) (adjusted p-value < 0.05). Additionally, log2-transformed and quantile-normalized counts of reads at significantly changing loci were used as input for differential motif analysis with GimmeMotifs.

#### Motif analysis

Motif analysis was performed using GimmeMotifs (Bruse and van Heeringen, 2018; van Heeringen and Veenstra, 2011). We used Gimme maelstrom with default settings to identify differential motif enrichment in ATAC-seq peaks. Motifs were filtered out if they could only be bound by factors that were not expressed in either genotype. For promoter region analysis, transcription start sites (TSS) were extracted using the bioMart package (Durinck et al., 2009). A 500 bp region around the TSS was used as input for gimme maelstrom. To define promoters that contained an RXR motif, we provided promoter regions of all expressed genes to gimme scan using a custom motif file that only contained the motif of interest. Visualization of motif analysis was done using the pheatmap package.

### Quantification and statistical analysis

For goblet cell quantification, we performed a Mann-Whitney U test using Prism 5. For organoid size quantifications, we performed an ANOVA followed by Dunetts post-hoc test to assess directionality using Prism 5. Where applicable, mean ± SD was plotted, n numbers are provided in figure legends.

## Data Availability

Sequencing data have been deposited at GEO and are publicly available as of the date of publication. Additionally, this paper analyzes existing, publicly available data. Accession numbers are listed in the key resources table. All other data reported in this paper will be shared by the lead contact upon request. This paper does not report original code. Any additional information required to reanalyze the data reported in this paper is available from the lead contact upon request.
