# Supplementary material for: Retinoic acid signaling drives differentiation toward the absorptive lineage in colorectal cancer
Source: iScience. 2021 Nov 15;24(12):103444. doi: 10.1016/j.isci.2021.103444 (PMC8633980; doi:10.1016/j.isci.2021.103444)
Supplement: Document S1. Figures S1–S3 [file mmc1.pdf]

iScience, Volume *24*

Supplemental information

Retinoic acid signaling drives differentiation toward the absorptive lineage in colorectal cancer

Roelof A. Wester, Lisa van Voorthuijsen, Hannah K. Neikes, Jelmer J. Dijkstra, Lieke A. Lamers, Siebren Frölich, Maarten van der Sande, Colin Logie, Rik G.H. Lindeboom, and Michiel Vermeulen

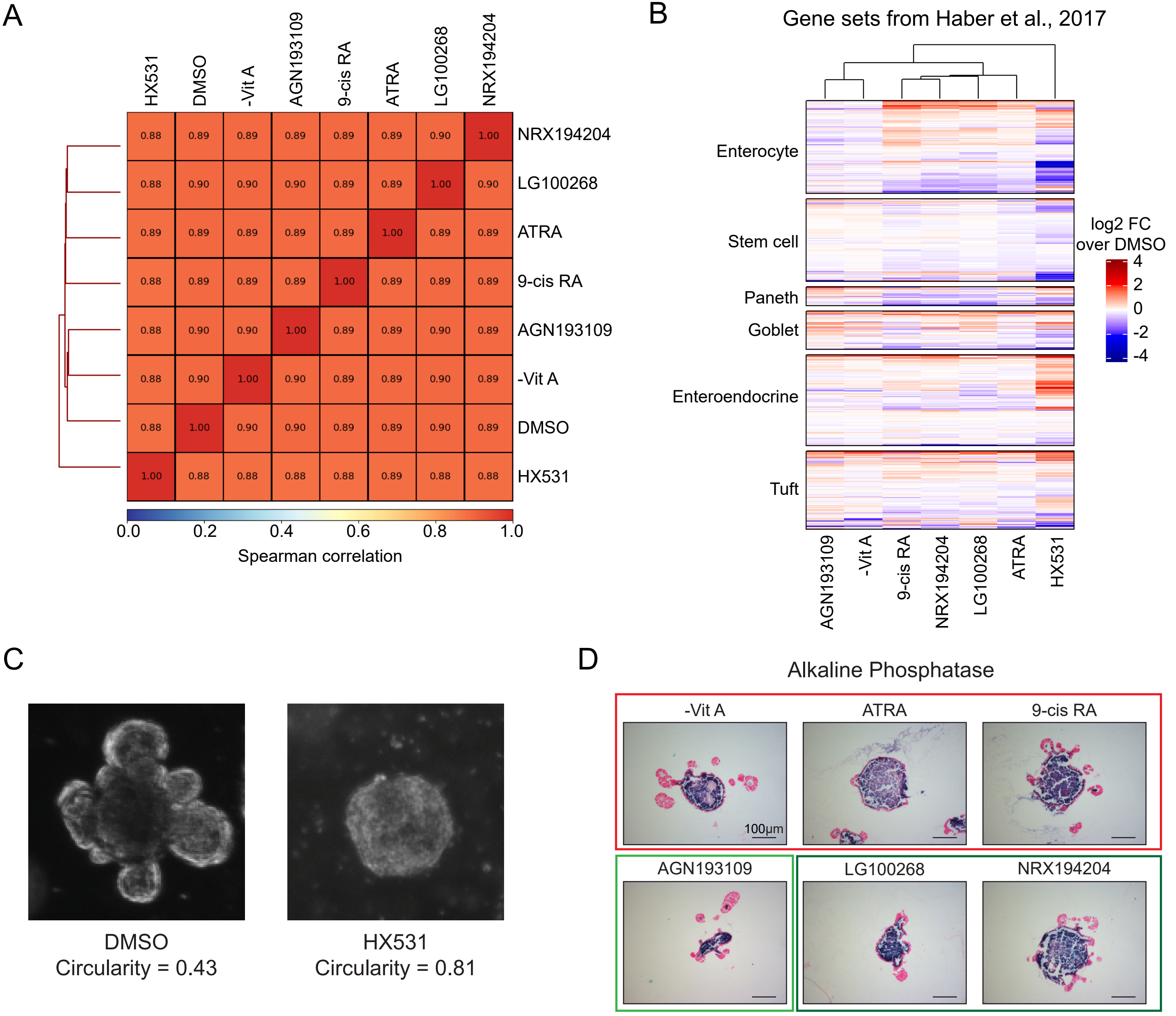
A
HX531
DMSO
-Vit A
AGN193109
9-cis RA
ATRA
LG100268
NRX194204
0.88 0.89 0.89 0.89 0.89 0.89 0.90 1.00 NRX194204
0.88 0.90 0.90 0.90 0.89 0.89 1.00 0.90 LG100268
0.89 0.89 0.89 0.89 0.89 1.00 0.89 0.89 ATRA
0.88 0.89 0.89 0.89 1.00 0.89 0.89 0.89 9-cis RA
0.88 0.90 0.90 1.00 0.89 0.89 0.90 0.89 AGN193109
0.88 0.90 1.00 0.90 0.89 0.89 0.90 0.89 -Vit A
0.88 1.00 0.90 0.90 0.89 0.89 0.90 0.89 DMSO
1.00 0.88 0.88 0.88 0.88 0.89 0.88 0.88 HX531
0.0 0.2 0.4 0.6 0.8 1.0
Spearman correlation
B
Gene sets from Haber et al., 2017
Enterocyte
Stem cell
Paneth
Goblet
Enteroendocrine
Tuft
log2 FC over DMSO
4
2
0
-2
-4
AGN193109
-Vit A
9-cis RA
NRX194204
LG100268
ATRA
HX531
C
DMSO
Circularity = 0.43
HX531
Circularity = 0.81
D
Alkaline Phosphatase
-Vit A
ATRA
9-cis RA
100μm
AGN193109
LG100268
NRX194204

Figure S1: RA perturbations in intestinal organoids, related to Figure 2

A) Spearman correlation matrix of all samples after filtering (Methods). Correlation coefficients are generally high, HX531-treated mouse small intestinal organoids cluster away from other samples.

B) Heatmap showing fold change compared to DMSO for cell-type specific gene sets in mouse small intestinal organoids treated with indicated compounds. Activation of RAR and RXR leads to increase in enterocyte genes, whereas inhibition leads to a decrease of enterocyte genes.

C) Example images of a DMSO and HX531-treated organoids with a low and high level of circularity, respectively.

D) Alkaline Phosphatase / Nuclear Fast Red staining for other used conditions. Despite observable changes at transcriptome level, these treatments do not result in reduced expression of alkaline phosphatase at the apical membrane or in the lumen of organoids. Colored rectangles refer to colors used in Fig1A.

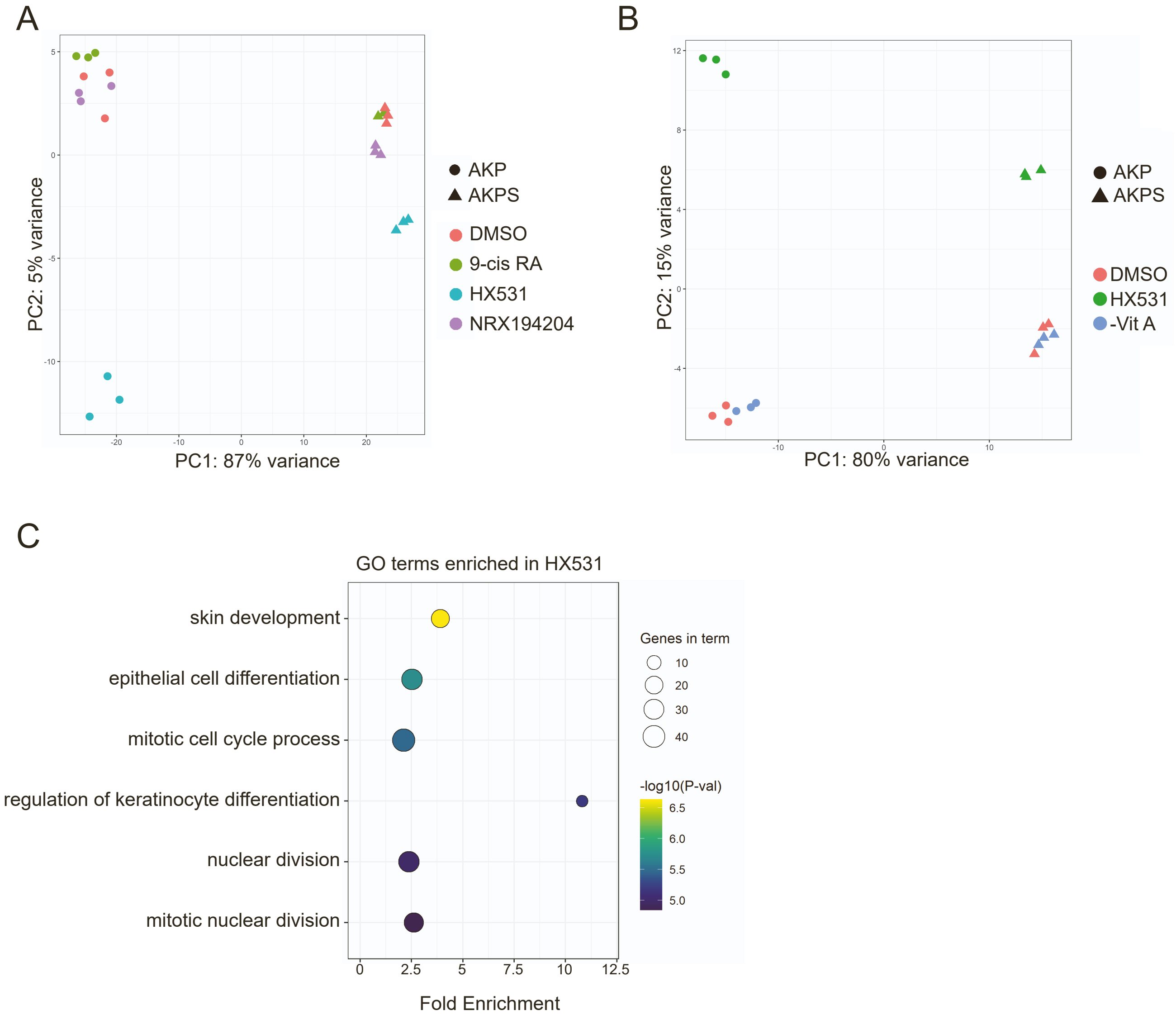
A
PC2: 5% variance
PC1: 87% variance
5
0
-5
-10
-20
-10
0
10
20
AKP
AKPS
DMSO
9-cis RA
HX531
NRX194204
B
PC2: 15% variance
PC1: 80% variance
12
8
4
0
-4
-10
0
10
AKP
AKPS
DMSO
HX531
-Vit A
C
GO terms enriched in HX531
skin development
epithelial cell differentiation
mitotic cell cycle process
regulation of keratinocyte differentiation
nuclear division
mitotic nuclear division
0
2.5
5
7.5
10
12.5
Fold Enrichment
Genes in term
10
20
30
40
-log10(P-val)
6.5
6.0
5.5
5.0

Figure S2: Colon organoid RNA-seq quality control, related to Figure 3

A) Principal Component analysis showing different genotypes (shape) and treatments (color). PC1 contains the majority of variance, and separates samples by genotype, PC2 separates samples based on treatment. 9-cis RA and NRX194204 both cluster with DMSO treated organoids of the corresponding genotype. HX531-treated organoids show global transcriptomic changes.

B) Principal Component analysis for different batch containing DMSO, DMSO – Vit A and HX531-treated organoids. Plot shows genotype (shape) and treatment (color). PC1 contains the majority of variance, and separates samples by genotype, PC2 separates samples based on treatment. DMSO and DMSO – Vit A samples cluster together, whereas HX531-treated organoids show global transcriptomic changes.

C) Bubbleplot showing GO terms enriched in HX531-treated organoids compared to DMSO-treated organoids. Fold enrichment is plotted on x-axis, p-value is indicated by color, and size of gene set by size. Terms enriched pertain to general epithelial development, or are specific to epithelial tissues that are not intestinal.

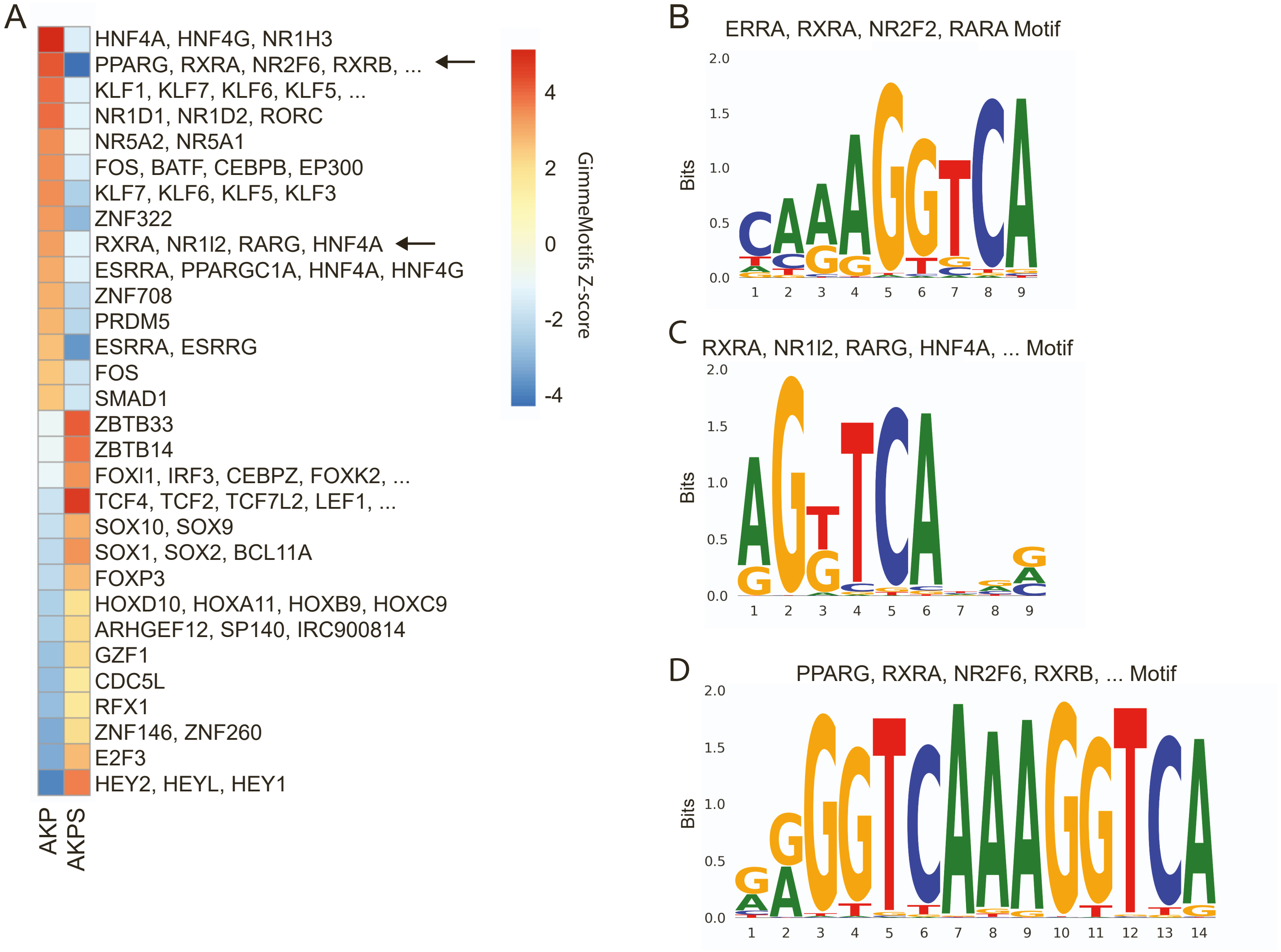
A
HNF4A, HNF4G, NR1H3
PPARG, RXRA, NR2F6, RXRB, ...
KLF1, KLF7, KLF6, KLF5, ...
NR1D1, NR1D2, RORC
NR5A2, NR5A1
FOS, BATF, CEBPB, EP300
KLF7, KLF6, KLF5, KLF3
ZNF322
RXRA, NR1I2, RARG, HNF4A
ESRRA, PPARGC1A, HNF4A, HNF4G
ZNF708
PRDM5
ESRRA, ESRRG
FOS
SMAD1
ZBTB33
ZBTB14
FOXI1, IRF3, CEBPZ, FOXK2, ...
TCF4, TCF2, TCF7L2, LEF1, ...
SOX10, SOX9
SOX1, SOX2, BCL11A
FOXP3
HOXD10, HOXA11, HOXB9, HOXC9
ARHGEF12, SP140, IRC900814
GZF1
CDC5L
RFX1
ZNF146, ZNF260
E2F3
HEY2, HEYL, HEY1
AKP
AKPS
GimmeMotifs Z-score
4
2
0
-2
-4
B
ERRA, RXRA, NR2F2, RARA Motif
Bits
C
RXRA, NR1I2, RARG, HNF4A, ... Motif
Bits
D
PPARG, RXRA, NR2F6, RXRB, ... Motif
Bits

Figure S3: Motif analysis in colon organoids, related to Figure 4

A) Heatmap showing enrichment of indicated motifs in the promoters of significant DE genes (p-value < 0.05) between AKP and AKPS. Promoter sequence was defined as 500bp around TSS, color indicates GimmeMotifs (maelstrom) Z-score. The 15 most enriched motifs in each line are shown, ranked according to z-score in AKP. Promoters of genes expressed in AKP contain differentiation-associated motifs such as HNF4A and HNF4G, as well as two motifs that can be bound by RXRA (indicated with arrows).

B) Sequence logo for the ERRA, RXRA, NR2F2, RARA motif indicated in Figure 4A. It shows great similarity to the consensus DR-half site sequence (AGGTCA).

C) Sequence logo for the RXRA, NR1I2, RARG, HNF4A… motif indicated in Figure S3A. It shows similarity to the consensus DR-half site sequence (AGGTCA).

D) Sequence logo for the PPARG, RXRA, NR2F6, RXRB… motif indicated in Figure S3B. It shows high similarity to two repeats of the DR-half site sequence with one nucleotide spacing (AGGTCANAGGTCA).
